# Electronic and Crystallographic Examinations of the Homoepitaxially Grown Rubrene Single Crystals

**DOI:** 10.3390/ma13081978

**Published:** 2020-04-23

**Authors:** Yasuo Nakayama, Masaki Iwashita, Mitsuru Kikuchi, Ryohei Tsuruta, Koki Yoshida, Yuki Gunjo, Yusuke Yabara, Takuya Hosokai, Tomoyuki Koganezawa, Seiichiro Izawa, Masahiro Hiramoto

**Affiliations:** 1 Department of Pure and Applied Chemistry, Tokyo University of Science, Noda 278-8510, Japan; 2 Institute for Molecular Science (IMS), National Institutes of Natural Sciences, and SOKENDAI, Okazaki 444-8787, Japan; 3 National Metrology Institute of Japan, National Institute of Advanced Industrial Science and Technology (AIST), Tsukuba 305-8568, Japan; 4 Industrial Application Division, Japan Synchrotron Radiation Research Institute (JASRI), SPring-8, Hyogo 679-5198, Japan

**Keywords:** organic semiconductor, doping, photoelectron yield spectroscopy, grazing-incidence X-ray diffraction, gap states, organic photovoltaic cell

## Abstract

Homoepitaxial growth of organic semiconductor single crystals is a promising methodology toward the establishment of doping technology for organic opto-electronic applications. In this study, both electronic and crystallographic properties of homoepitaxially grown single crystals of rubrene were accurately examined. Undistorted lattice structures of homoepitaxial rubrene were confirmed by high-resolution analyses of grazing-incidence X-ray diffraction (GIXD) using synchrotron radiation. Upon bulk doping of acceptor molecules into the homoepitaxial single crystals of rubrene, highly sensitive photoelectron yield spectroscopy (PYS) measurements unveiled a transition of the electronic states, from induction of hole states at the valence band maximum at an adequate doping ratio (10 ppm), to disturbance of the valence band itself for excessive ratios (≥ 1000 ppm), probably due to the lattice distortion.

## 1. Introduction

Impurity doping is a key technology that boosts the functionalities of semiconductors. This is true not only for conventional inorganic semiconductors; in fact, a monumental work on bromine-doped perylene enkindled the dawn of “organic semiconductor” research [1,2] and led to the discovery of conductive polymers [3], charge transfer complexes [4,5], and molecular superconductors [5,6,7,8]. Although ordinary organic (opto-)electronic devices have been designed using intrinsic organic semiconductors without any intentional doping, on-demand introduction of dopants into molecular semiconductor materials is a long-standing topic in this research field (for review, see e.g., [9,10,11,12,13,14]).

**Figure 1:**
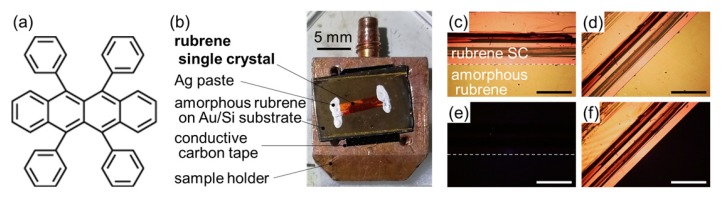
(**a**) Molecular structure of rubrene. (**b**) Photograph of a homoepitaxial rubrene sample for photoelectron yield spectroscopy (PYS) measurements. (**c**,**d**) Optical micrographs of the sample shown in (**b**). (**e**,**f**) Crossed-nicols polarized micrographs at the same sample geometries as (**c**,**d**), respectively. The scale bars in (**c**–**f**) correspond to 0.5 mm. The edge of the rubrene single crystal is highlighted with dashed lines in (**c**,**e**).

Unlike the case of silicon, controlled doping in organic semiconductor single crystals with minimized lattice distortion and phase separation has been a serious challenge. Recently, Ohashi and coworkers proposed a novel methodology for the production of organic semiconductor single crystals including dopant molecules [15]. This technique is based on molecular beam epitaxy, where an organic semiconductor species is deposited on a single crystal substrate of the same kind of molecule (i.e., homoepitaxy) in vacuo [16,17]. Through rigorous tuning of the evaporation rates of the ‘host’ molecule and the dopant, introduction of FeCl_3_ molecules as an acceptor into the bulk crystal lattice of rubrene [Figure 1a] at accurately controlled concentrations down to the weight ratio of 1 ppm and of uniform spatial distributions was successfully achieved in that work [15]. Moreover, a single crystal homojunction was fabricated by doping of donor and acceptor molecules in either side of the homoepitaxial rubrene single crystal, and it was actually shown to exhibit a photovoltaic response [18]. 

In the present study, homoepitaxially grown single crystalline overlayers of rubrene were examined by means of photoelectron yield spectroscopy (PYS) and grazing-incidence X-ray diffraction (GIXD). Rubrene is a representative of small-molecule *p*-type organic semiconductors and is known to exhibit remarkably high mobility of conductive holes in its single crystal phase [19,20,21], which is understood in the band transport framework [22,23]. PYS is a suitable methodology for electronic characterization of specimens of low electric conductance [24,25], which is the case for intrinsic rubrene as a wide-gap (2.8 eV [26]) semiconductor, and particularly for detecting small modifications at the highest-occupied electronic states in an extreme sensitivity [27,28,29]. GIXD is a technique for determination of the crystal structures of thin films, and has successfully been applied to hetero-epitaxial organic semiconductor junctions built on molecular single crystals for specification of the lattice orientations [30,31,32] and even for evaluation of their crystallographic qualities by using high-resolution apparatuses [33,34,35]. It was confirmed that homoepitaxial rubrene single crystals grow without any apparent lattice distortion up to the thickness of 100 nm by the present GIXD measurements. In addition, high-sensitivity PYS analyses clearly unveiled evolution of the electronic structures of the homoepitaxial rubrene upon increase in the doping ratio of FeCl_3_; that is, induction of the holes at the top of the valence band in the initial stage was followed by disturbance of the valence band for excessive doping ratios. 

## 2. Materials and Methods 

Single crystals of rubrene were produced by a horizontal physical vapor transport (PVT) technique [36]. Details of the PVT equipment used in this work can be found elsewhere [37]. The obtained crystals were subsequently bounded on Au-coated Si wafer pieces using conductive Ag paste for the electronic measurements [Figure 1b–f], or on Si pieces covered with the native oxide by the electrostatic force for the crystallographic analyses, to prepare “substrates”. The homoepitaxial rubrene overlayers were produced by vacuum deposition on the rubrene single crystal samples at a low evaporation rate of ca. 3 pm/s by using a vacuum chamber built in a glove box [15]. Bulk-doped homoepitaxial rubrene single crystal samples were fabricated by simultaneous deposition of FeCl_3_ as a *p*-type dopant together with rubrene onto the single crystal rubrene surfaces. For doping rates of less than 1000 ppm, rotating disks with various aperture ratios (e.g., 10:1 and 100:1 for the doping ratios of 100 ppm and 10 ppm, respectively) were interposed between the FeCl_3_ evaporation source and the samples, while the evaporation condition at the origin was maintained as that for 1000 ppm doping [15]. 

The crystallinity of the homoepitaxial rubrene samples was evaluated by GIXD at BL46XU of SPring-8. The X-ray wavelength and glancing angle were set at 0.100 nm and 0.12°, respectively. Overall diffraction patterns of the sample surfaces were collected by two-dimensional (2D) GIXD measurements by using a 2D X-ray detector PILATUS300K, which was set approximately perpendicular to the incident X-ray, and crystallographic coherent lengths along in-plane directions were evaluated by high-resolution (HR) GIXD spot analyses in the 2θ directions using a NaI scintillation counter and a Ge(111) analyzer crystal. Detailed descriptions of the experimental conditions are found elsewhere [30,35]. The GIXD analyses were conducted in the ambient atmosphere. 

The electronic states of the homoepitaxial rubrene samples were analyzed by using a home-built PYS apparatus [29]. For the PYS analyses, exposure of the samples to the ambient atmosphere was thoroughly avoided by the following procedures: (1) the rubrene single crystal “substrates” recrystallized in a purified nitrogen steam were directly conveyed to a glove box filled with a nitrogen atmosphere, (2) the rubrene single crystal substrates were prepared in the glove box and were transferred by using an air-tight container filled with nitrogen and a de-oxidation agent, (3) the samples were introduced into the aforementioned glove box equipped with the vacuum chamber, (4) the homoepitaxial rubrene overlayers were deposited up to the overlayer thickness of 50 nm, (5) the samples were transferred back to the first glove box, and (6) the samples were introduced to the ultra-high vacuum system for PYS measurements by using a vacuum vessel. 

## 3. Results

### 3.1. Crystallographic Analyses

**Figure 2:**
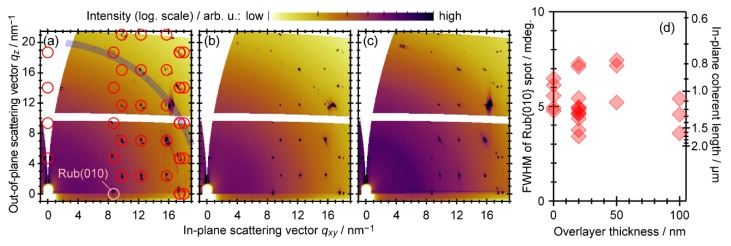
(**a–d**) Two-dimensional grazing incidence X-ray diffraction (2D-GIXD) images of rubrene single crystal samples (**a**) without and with the homoepitaxially grown rubrene overlayers of (**b**) 50 nm- and (**c**) 100 nm-thick. These images are integrated for the sample azimuthal angle over 180° (900 images taken for every 0.2°). Expected positions for the diffraction spots from the (100) surface of rubrene single crystal and Si (powder) are indicated with circle marks and a thick arc, respectively, on (**a**). (**d**) Full-width at half-maximum (FWHM) in 2θ direction of the {010} diffraction spots of rubrene [denoted as Rub{010}] collected by high-resolution grazing-incidence X-ray diffraction (HR-GIXD) measurements plotted as a function of the rubrene overlayer thickness. The corresponding crystallographic coherent length (up to 2 μm) estimated from the Scherrer equation is also indicated in the right axis for reference.

Figure 2a shows the two-dimensional (2D) X-ray diffraction pattern of a rubrene single crystal sample obtained by integration of 2D-GIXD images taken during continuous rotation of the in-plane azimuthal angle ϕ of the sample over 180°. The diffraction spots seen in this image were reproduced by a simulated pattern, as indicated with circle marks, for the (100) surface assuming a known crystal structure of rubrene [38], whereas diffraction ascribable to Si powder (blue arc around |***q***| = 20.04 nm^−1^ [39]) probably originated from the wafer piece that the sample was fixed on. ϕ-integrated 2D-GIXD images taken on rubrene single crystals with 50-nm- and 100-nm-thick overlayers of rubrene are displayed as Figure 2b,c, respectively. While increase in the background intensity (|***q***| < 8 nm^−1^) was observed to some extent for the 100-nm-thick overlayer as seen in Figure 2c, the obtained diffraction patterns agreed with that of the bare rubrene single crystal, irrespective of the thickness of the rubrene overlayer. It has been reported that rubrene can be crystallized in different polymorphs, triclinic and monoclinic phases, from solution [40,41], and one theoretical work has predicted that the triclinic phase is more stable than the bulk orthorhombic phase [42]. However, emergence of the different polymorphs can be negligible or non-existent, as no sign of diffraction spots attributable to the triclinic or monoclinic phase was detected in the present 2D-GIXD images.

The ϕ-dependence of the 2D-GIXD results indicated that diffraction intensity at ***q*** ≡ (*q_xy_*, *q_z_*) = (8.74 nm^−1^, 0 nm^−1^), which corresponds to {010} diffraction spots for the (100) surface of the rubrene single crystal, appeared only for specific ϕ geometries in a 180° periodicity, as expected from the symmetry of the crystal lattice structure. Other spots, e.g., the {111} spots at (*q_xy_*, *q_z_*) = (9.76 nm^−1^, 2.34 nm^−1^)], were also confirmed to come out at expected ϕ-intervals for all samples. These results corroborated homoepitaxial growth of the rubrene overlayer by complete matching of the crystallographic orientation with the underlying rubrene single crystals. 

Figure 2d shows full-width-at-half-maxima (FWHM) of 2θ-profiles for the Rub{010} diffraction spots collected by HR-GIXD measurements plotted as a function of the thickness of the homoepitaxial rubrene overlayers. Individual marks correspond to the data obtained at various sample orientations from several samples for each thickness. The spot width is formally related to a crystallographic coherent length (or mean crystallite size) through the Scherrer equation, which is also indicated on the right axis of Figure 2d for reference. Since the actual crystalline domain size of the rubrene single crystals used in this study was at least several-mm-wide, as exemplified in Figure 1, the “coherent size” for the 0-nm-thick sample (i.e., bare rubrene single crystals) does not give any reasonable dimensions of the sample but has to be considered to be restricted by angular resolution of the present experimental setup [43]. The present results suggest an absence of structural deterioration, at least in a scale of this size, upon growth of the homoepitaxial rubrene crystals to the thickness of 100 nm. Therefore, it should be concluded that, at least in terms of structure, the homoepitaxial rubrene overlayers and the bulk single crystal rubrene underneath are identical, not distinguishable as previously suggested [15]. 

### 3.2. Electronic Analyses

**Figure 3:**
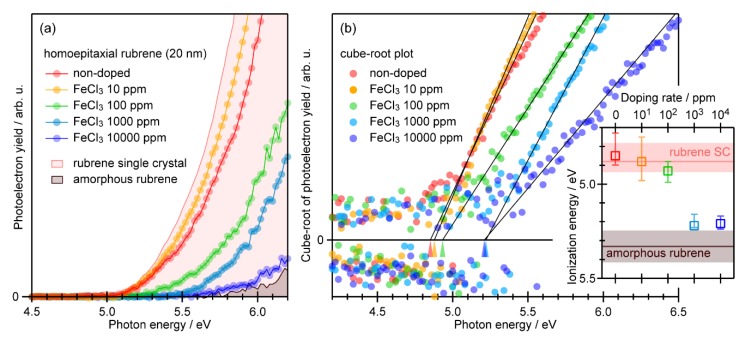
(**a**) PYS spectra of 20-nm-thick homoepitaxial rubrene overlayers of various doping ratios grown on rubrene single crystal substrates. PYS spectra of a single crystal sample and an amorphous film of rubrene are also displayed as hatched areas. (**b**) PYS spectra plotted in the cube-root scale of the photoelectron yield. Ionization energy positions derived from least-squares fitting of these spectra are indicated with wedge marks, where thin lines show the fitting results. (Inset) Ionization energy of rubrene overlayers plotted as a function of the doping rate. The ionization energy values for the bare rubrene single crystal (SC) and the amorphous rubrene are also indicated with pink and brown lines, respectively, where light-colored bands indicate error ranges.

The variation in PYS spectra of 20-nm-thick homoepitaxial rubrene overlayers depending on the FeCl_3_ doping ratio is presented in Figure 3a. The spectra of a bare rubrene single crystal and an amorphous rubrene thin film (thickness of 20 nm) grown on an indium-tin-oxide substrate are also displayed. While the homoepitaxial overlayers without FeCl_3_ doping and with 10 ppm FeCl_3_ exhibited substantially the same trends of photoelectron yield *Y* versus photon energy *h*ν as that of the bare rubrene single crystal, increase in the doping ratio led to a rightward shift of the spectra approaching that of the amorphous rubrene. 

The photoelectron yield from organic molecular solids can be approximated with the following empirical cube law as a function of *h*ν [24,29,44,45,46]: $Y\left(h\upsilon \right)=A{\left(h\upsilon -I_{s}\right)}^{3}\cdot S\left(h\upsilon -I_{s}\right)$, where *I*_s_ is the ionization energy of the sample, *A* is a material dependent parameter relating to the photoemission cross section, and *S*(*x*) is an adequate step function that switches from zero to unity when *x* goes from negative to positive. This formulation is valid only in a range where (*h*ν − *I*_s_) is not too great [46]. The *I*_s_ values for the bare rubrene single crystal and amorphous rubrene samples were derived by least-squares fitting of the spectra as ($4.88_{\;-\;0.09}^{\;+\;0.06}$) eV and (5.33±0.08) eV, respectively, which is in good accordance with previous PYS [45] and ultraviolet photoelectron spectroscopy (UPS) works [47,48,49,50,51,52]. For *h*ν > *I*_s_, this formula can be transformed into: ${\left[Y\left(h\upsilon \right)\right]}^{1/3}=A^{\prime }\left(h\upsilon -I_{s}\right)$. This means that a *x*-intercept of a linear onset for the [*Y*(*h*ν)]^1/3^ plot corresponds to *I*_s_ of each sample. In Figure 3b, the PYS spectra of the 20-nm-thick homoepitaxial overlayers given in Figure 3a are replotted in the [*Y*(*h*ν)]^1/3^ scale. The *I*_s_ values depending on the FeCl_3_ doping ratio are plotted on the inset graph, where the error bars indicate possible ranges covering variation of the results depending on the fitting conditions and sample individuals. This suggests a jump in *I*_s_ when the doping ratio was increased from 100 ppm to 1000 ppm. 

## 4. Discussion

The ionization energy of organic semiconductors is not material specific but depends largely on molecular packing in the solid state [53]. In the case of rubrene, it was proposed that the difference in *I*_s_ between single crystals and amorphous films should be attributed mostly to formation of the inter-molecular electronic “bands” for the former [45]; i.e., occurrence of the energy dispersion upshifts the upper edge of the highest occupied electron energy in comparison to the original discrete molecular orbital position. Actually, the formation of the highest-occupied molecular-orbital (HOMO) band (valence band) of ca. 0.45 eV-wide was demonstrated for the single crystal rubrene [48,49,54], and a narrower photoemission linewidth of the HOMO peak was observed for the amorphous rubrene films, suggesting an absence of band dispersion [55]. These suggest correspondence between the *I*_s_ magnitude and the crystalline order of rubrene. 

As indicated in Figure 3, the PYS spectra and the *I*_s_ values for the non-doped and 10-ppm-doped samples revealed good accordance with those of the rubrene single crystal itself, which corresponds to the fact that these homoepitaxial rubrene overlayers exhibit good crystallinity. On the other hand, *I*_s_ for the 1000-ppm-doped and 10000-ppm-doped rubrene overlayers approached the value for amorphous rubrene, which should be ascribed to structural disordering due to the presence of significant amount of the dopant molecules as previously suggested by atomic force microscopy (AFM) results [15]. The case for the 100-ppm-doped rubrene may be an intermediate. In the previous work [15], while the AFM images did not exhibit any signs of distortion up to this doping ratio, the Hall mobility decayed significantly in increasing the doping ratio from 10 ppm to 100 ppm suggesting an emergence of lattice disturbances as scattering centers. The present *I*_s_ position for the 100-ppm-doped sample was close to that of the bare rubrene single crystal, which implies that the lattice of the homoepitaxial rubrene was not largely disturbed by the presence of 100 ppm FeCl_3_. However, this was not entirely free from the structural distortion that may diminish the photoelectron yield from the ‘well-crystalized’ rubrene and/or slightly reduce the *I*_s_ value. 

**Figure 4:**
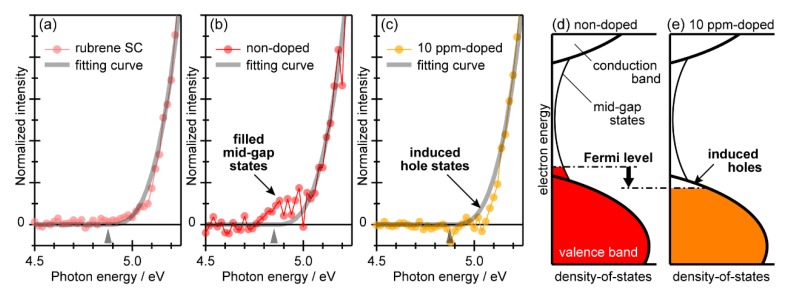
(**a–c**) Magnified PYS spectra of the (**a**) bare rubrene single crystal, (**b**) non-doped homoepitaxial rubrene overlayer, and (**c**) 10-ppm-doped homoepitaxial rubrene. The vertical scales of each graph are normalized by the intensity factor “*A*” of the cube-root function. The fitting curves and estimated ionization energy positions for the respective spectra are displayed as thick gray curves and gray wedge marks, respectively. (**d**,**e**) Schematic drawings of the expected electronic states of the (**d**) non-doped and (**e**) 10-ppm-doped homoepitaxial rubrene samples.

Whereas the three PYS spectra in Figure 3 for the bare rubrene single crystal, non-doped homoepitaxial rubrene, and 10-ppm-doped sample resemble each other, small but significant differences were found when taking a closer look at the photoemission threshold regions. As shown in Figure 4a, the spectra for the bare rubrene single crystal rose along the cube-law curve on the whole. It is noteworthy that the previously reported PYS and UPS spectra for the single crystal rubrene exhibited photoemission signals, even in the energy region beyond the main valence band edge determined by the cube-law fitting [48], and that feature was ascribed to the so-called “oxygen-related band gap state” that had been found on the rubrene single crystal samples being exposed to air [56]. Since the present samples kept their surfaces ‘fresh’ by the thorough avoidance of being exposed to the ambient conditions before the PYS experiments, the spectral profile given in Figure 4a, and thus the cube-law curve, can be considered as an archetype of the *Y*(*h*ν) pattern for the intrinsic valence band of the single crystal rubrene. On the other hand, the spectra of the non-doped homoepitaxial rubrene exhibited extra intensities in the low-energy side of the cube-law curve, as shown in Figure 4b. This means that some electrons did exist even though that energy range was within the band gap beyond the valence band onset. In contrast, for the 10-ppm-doped samples, the photoelectron yield only at the vicinity of the valence band edge was shaved off from the expected cube-law curve, which suggests that electrons accommodating at the valence band maximum (VBM) were taken away. 

The *Y*(*h*ν) magnitude in principle reflects the occupied density-of-states (DOS) of the specimen above that energy (*h*ν) position with respect to its vacuum level. While a conclusive understanding about a formulation for deducing the accurate DOS distribution from the PYS spectrum [25,57,58,59] has not yet been reached, nevertheless, characteristics of the electronic structures of the non-doped and 10-ppm-doped homoepitaxial rubrene can be outlined as Figure 4d,e, respectively. The additional photoemission feature for the non-doped homoepitaxial rubrene indicates the emergence of occupied electronic states tailing into the energy gap range. Note that this sample was not exposed to air either; the “oxygen-related” should be excluded from a possible origin for these mid-gap states. Instead, like the Urbach tail of inorganic semiconductor materials [60], the presence of slight structural disordering in the homoepitaxial overlayers presumably caused the rise of the DOS in the energy gap region. Even though the crystallographic structure of the homoepitaxial overlayer is identical to the rubrene single crystal substrate, it was proposed that the growth in vacuo of the homoepitaxial rubrene is not at the thermodynamic equilibrium, and thus the growth stress and strain has to be present in the interior of the homoepitaxial rubrene films [17]. Actually, relatively low Hall mobility (0.2 cm^2^V^−1^s^−1^) for the non-doped homoepitaxial rubrene crystals [15], in comparison to that of bare rubrene single crystal samples (~0.6 cm^2^V^−1^s^−1^) measured by the same group [61], implies the existence of latent structural disturbance playing a role in the scattering centers for charge carriers. P-type doping to semiconductors generally pulls the Fermi level down to the deeper electron binding energy side. In fact, it was reported that the work function of amorphous rubrene films increased from 4.69 eV to 5.02 eV upon doping of 10 ppm FeCl_3_ [15]. In the present homoepitaxial rubrene case, the doping of 10 ppm FeCl_3_ made little impact on the crystal lattice, and thus on the main valence band, but only shifted the Fermi level downwards, which swept the electrons in the mid-gap states out and induced the holes at the top of the valence band. 

## 5. Conclusions

The homoepitaxial overlayers of rubrene grown on the single crystal rubrene were examined by GIXD and PYS in terms of their crystallographic qualities and electronic structures, respectively. The GIXD results confirmed the absence of apparent structural disturbance, as far as the present resolution limit, for the homoepitaxial rubrene single crystals up to the thickness of 100 nm. The PYS results indicated that the ionization energy of the main valence band edge of the homoepitaxial rubrene is identical to that of the rubrene single crystal itself, that doping of FeCl_3_ up to 10 ppm hardly affects the main valence band, and that excessive doping over 1000 ppm leads to the transition of the electronic states to those of the amorphous phase of rubrene. High-sensitivity analyses of the PYS revealed the emergence of the filled mid-gap states above the VBM of the non-doped homoepitaxial rubrene and the induced hole states at the VBM upon doping of the 10 ppm FeCl_3_.
